# The Sutureless Excimer Laser Anastomosis Clip Pilot Study: a feasibility and safety study

**DOI:** 10.1007/s00701-022-05182-1

**Published:** 2022-05-07

**Authors:** Albert van der Zwan, Kiki Gortzak, Bart de Boer, Saskia Redegeld, Sander van Thoor, Cornelius Tulleken

**Affiliations:** 1 Department of Neurosurgery, University Medical Center Utrecht, Brain Technology Institute, Utrecht, The Netherlands; 2 Brain Technology Institute, Utrecht, The Netherlands; 3 Department of Neurosurgery, St. Elisabeth Hospital, Brain Technology Institute, Utrecht, Tilburg The Netherlands

**Keywords:** Anastomosis, Non-occlusive Sutureless Technique, Clip, Bypass

## Abstract

**Background:**

The excimer laser-assisted non-occlusive anastomosis (ELANA) bypass technique may have the advantage of its non-occlusive design in the treatment of last-resort cases where endovascular treatment or direct clipping is considered to be unsafe. However, the technique remains technically challenging. Therefore, a sutureless ELANA Clip device (SEcl) was developed to simplify the technique avoiding tedious anastomosis stitching in depth.

The present study investigates the clinical feasibility and safety of the SEcl technique.

**Methods:**

Three patients with complex and large aneurysms in the anterior circulation were selected after multidisciplinary consensus that the aneurysms were too complex for endovascular or direct clipping treatment options. Bypass surgery was considered as a last-resort treatment option, and after preoperative evaluation and informed consent, SEcl bypass surgery was performed. Applicability, technical aspects and patient outcomes are assessed.

**Results:**

All aneurysms were excluded from the circulation. The creation of the intracranial anastomosis was easier and faster. No device-related serious adverse events were encountered, and all outcomes were favorable (one patient stable Modified Rankin Scale, two patients improved).

**Conclusion:**

The SEcl anastomosis technique is feasible and, considering the severity of the disease, relatively safe. It can be considered a treatment option in very difficult-to treat last-resort aneurysm cases. From this study, further developments in minimizing clip size and application in cardiac surgery are initiated.

**Supplementary Information:**

The online version contains supplementary material available at 10.1007/s00701-022-05182-1.

## Introduction

In the treatment of very large and complex intracranial aneurysms, both conventional bypass techniques utilizing temporary recipient vessel occlusion and the excimer laser-assisted non-occlusive anastomosis (ELANA) bypass technique are possible strategies when endovascular methods are not safely applicable [11, 17]. The potential advantage of the ELANA technique is the absence of temporary occlusion of major arteries, decreasing the risk of intraoperative ischemia.

Despite the advantage of omitted occlusion time, the non-occlusive ELANA bypass technique remains technically challenging, with several micro-sutures to be performed on the recipient vessel at depth within a small craniotomy window. It requires considerable skills from the neurosurgeon to connect the donor graft to the recipient vessel with micro-sutures, most often at the intracranial internal carotid artery (ICA) [20]. This procedure, even in experienced hands, is time-consuming and takes a minimum of 60 min for each anastomosis to be completed.

This created the need for simplification of the technique. Reducing the number of sutures needed in the depth could simplify the procedure and reduce surgery time and complication risk. Adapting the technique to a sutureless device was therefore a logical next step in the continuous search for improvement of the technique. This could also enable more neurosurgeons to include the technique in their surgical repertoire. The first attempt to achieve this was the development of the sutureless ELANA slide (SEsl) technique that after numerous preclinical tests in three models (in vitro, acute in vivo and long-term in vivo) was found to be equivalent to the conventional ELANA technique [18]. In the first patient treated with this device, the creation of the bypass and the occlusion of the aneurysm were successful, and the patient was postoperative well. However, a pseudoaneurysm developed as shown with digital subtraction angiography (DSA) 14 days postoperative which resulted in a hemorrhagic event. The cause of this was the force that was needed to slide the device into the intracranial donor vessel. Since then, the search for a safer simplification of the ELANA technique has continued and the sutureless ELANA Clip technique (SEcl) was developed [4–6].

**Fig. 1:**
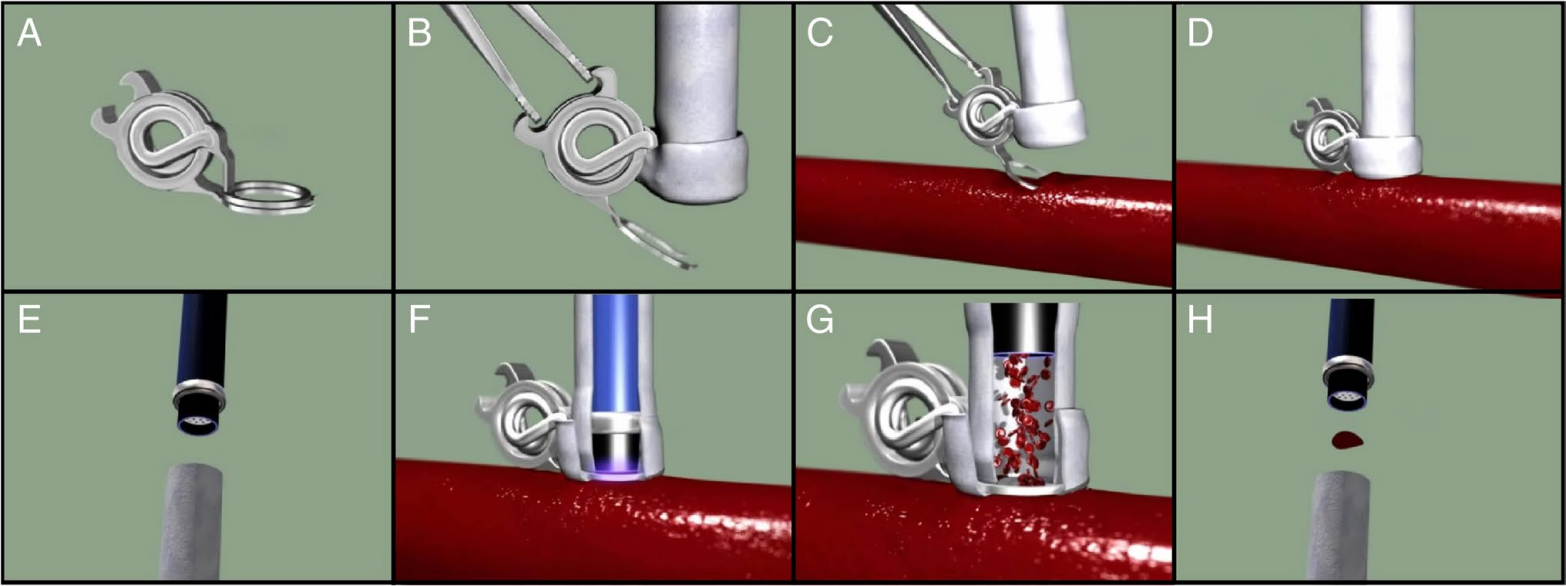
**a**: Sutureless ELANA clip; **b**: sutureless ELANA clip with mounted vein; **c**: insertion into vessel; **d**: closure of clip; **e**: inserting laser catheter; **f**: lasering anastomosis; **g**: retrieval of catheter; **h**: retrieval of flap

By introducing a spring at the back of the SEsl that connected the ring to the pins, the device was enabled to open at insertion and lock itself onto the recipient vessel at closure avoiding the forces of friction during application (Fig. 1, Video 1, Supplemental Digital Content 1). This might reduce the risk of pseudoaneurysm formation as no friction is applied.

The conclusion of these preclinical studies was that the SEcl technique is not inferior to the ELANA technique regarding safety, patency, flap retrieval rate, flow, endothelialization and long-term remodeling. Based on these studies, the significantly shorter application time and superior hemostasis, the SEcl technique could be preferable over the ELANA technique. A pilot study in patients is the next step based on our extensive preclinical studies. The technique will be tested as a last-resort treatment modality for complex aneurysms when endovascular treatment or direct surgical clipping is considered to be an unsafe option.

The positive findings from our extensive preclinical assessment needed to be confirmed in a pilot study to clinically assess the feasibility and safety of the technique and the use of this device.

Initially, the technique will be tested as a last-resort treatment modality for complex aneurysms when endovascular treatment or surgical clipping is not an option.

## Methods

### Study design

The sutureless ELANA Clip pilot study was a phase 2 single-center one-armed pilot study tested as last-resort treatment option for patients with complex aneurysms of the anterior cerebral circulation. The study protocol was approved by the central medical ethics committee and the research board of the University Medical Center Utrecht (13–655/D, UMC Utrecht).

Diagnosis had to have been confirmed by computed tomographic angiography (CTA), magnetic resonance angiography (MRA) and digital subtraction angiography (DSA). Balloon occlusion test (BOT) was performed when applicable. Before possible inclusion, each patient was discussed in our weekly multidisciplinary meeting attended by vascular neurologists, endovascular radiologists and vascular neurosurgeons to evaluate the indication for ELANA high-flow bypass and to rule out other means of treatment such as endovascular ICA occlusion, stenting or coiling. When no other treatment than bypass surgery was considered safe enough, additional investigation of the applicability of one of the large saphenous veins (VSM) as an ELANA donor graft was performed. Doppler was used for estimating the absence of varicose, stenosis or obliteration. Also, the length of the open VSM was estimated to ensure its length for bridging the distance from the proximal to distal anastomosis. When the ELANA technique was deemed feasible as a treatment option, the patient was informed about the team’s advice to perform an ELANA procedure and that participation in the sutureless ELANA Clip pilot trial was possible. Detailed information about the Clip pilot study, including operative risks, was extensively provided both orally and in writing to the patient before enrollment in the sutureless ELANA Clip Trial.

All patients provided written informed consent before inclusion. The study was supervised by an external independent Data Safety Monitoring Board (DSMB) and monitored by the Julius Center UMC Utrecht (the Netherlands).

## Patient inclusion

Inclusion criteria were defined for patients ≥ 18 years old on the date the informed consent form was signed and a temporary or permanent high-flow ELANA bypass in the treatment of a large or giant aneurysm in the anterior circulation was needed as determined by the above-mentioned multidisciplinary board.

Intraoperatively, patients could still be excluded from the trial and converted to a conventional ELANA bypass or conventional occlusive bypass operation if recipient vessels would be considered not suitable for the sutureless ELANA Clip bypass technique such as uncontrollable spasms in the graft and unexpected arteriosclerosis of the recipient vessels.

## Bypass procedure

As in historical ELANA procedures, carbasalate calcium (1dd 100 mg) medication was started 3 days prior to the planned surgery and continued postoperatively. When intraoperatively the flow through the bypass is low (as determined by the physician), 3000 IE of heparin (IV) might be administered once. The patient was fixed in the frame in supine position. Depending on the location and anatomy of the aneurysm, the incision and pterional approach were planned and performed. While the recipient artery was prepared at the prospective anastomosis side with minimal manipulation, the VSM was simultaneously harvested over a long trajectory (± 20 cm) and then microscopically prepared for usage as graft on a separate operating table. Eventual side branches were closed off using micro-hemoclips.

The prepared donor vessel was mounted by then inserting and folding the VSM over the opened ring of the SEcl and attached to itself with 2 or more micro-sutures (Prolene 8.0). A catheter sizer was inserted into the donor vessel to ensure adequate caliber for subsequent smooth insertion of the laser catheter. The clip was then gently slid into the artery using the clip applicator. On closure, the clip locked itself, fixating the donor onto the recipient artery. The SEcl device was now in place.

The laser catheter was then attached to the vacuum pump and the excimer laser apparatus, with a calibrated energy output of 10mJoules. The laser catheter was inserted and passed down the donor until it touched the recipient artery wall. The vacuum pump was switched on for 2 min to ensure adequate contact between the laser catheter fibers and the artery wall. The ELANA Laser (Spectranetics CVX300 Laser (Spectranetics, Colorado Springs, USA)) was then activated, creating the arteriotomy by excising a disk of artery wall (the flap). Laser settings were 2 × 5-s laser activation at 40 pulses/second with an interval of 5 s reset time. A temporary occlusion clip was placed on the donor approximately 3 cm from the anastomosis.

The proximal, extracranial anastomoses were conventionally constructed using a 4.2 mm punch for the arteriotomy and running sutures (Prolene 6.0). Finally, the end-to-end vein anastomosis is constructed using running sutures (Prolene 8.0) with heparin solution inserted in the bypass before temporal closure. The SEcl anastomosis time was defined as the time between the start of mounting the VSM to the clip and the end of lasering. After checks on bypass patency using flowmetry (Transonic Systems, USA) and indocyanine-green angiography (ICG), the internal carotid artery was definitively occluded using Mersilene 1.0.

## Outcome and safety measure

The primary outcome was the score on the modified Rankin scale at 90 days.

Secondary outcomes included modified Rankin score (MRS) at 7 days and 30 days postoperatively and at last follow-up. Imaging outcomes included bypass patency measured with CTA and/or DSA at 24 h and CTA and/or DSA or at 7 days and after 90 days.

We defined a favorable outcome as a successfully treated aneurysm and a better or equal postoperative MRS compared to the preoperative MRS score.

Safety variables included hemorrhagic complications, the progression of ischemic stroke or new ischemic stroke and death. If neurological deterioration developed perioperatively, additional neuroimaging was performed. All complications that occurred perioperatively, in the first 30 days, were recorded and registered as adverse events.

## Clinical and radiologic assessment

All patients underwent clinical assessment (including extensive neurological examination) at baseline (preoperatively), after 24 h, at 7 days or at discharge if earlier and at follow-up. Long-term follow-up was assessed on clinical basis.

Follow-up CTA and DSA were assessed for bypass patency, occlusion of the aneurysm, the presence of intracranial hemorrhage, ischemic infarction and other abnormalities.

## Statistical analysis

This pilot study functions as a clinical feasibility study. This first clinical experience with the novel sutureless ELANA Clip technique and study outcomes were therefore chosen to be presented descriptively on a case-by-case basis.

## Results

A total of three patients was recruited to participate in this study (Table 1).

**Table 1:** Patient Characteristics of the Sutureless ELANA Bypass Clip Trial. MRS: modified Ranking Scale, ECA: external carotid artery, ICA: internal carotid artery, MCA: middle cerebral artery

Characteristics	Patient 1	Patient 2	Patient 3
**Age**	61	62	66
**Sex**	Female	Female	Female
**Presenting symptom**	Subacute headache and vomiting, complete right n. III palsy (partial right n. IV and n. VI paresis)	Cranial n. III palsy right: diplopia, ptosis (OS > OD), visual disturbances	Cranial n. VI palsy right, diplopia
**Bypass Indication**	Giant right carotid siphon aneurysm (diameter 28 mm)	Giant right ICA aneurysm (diameter 30 × 28 mm)	Giant ICA aneurysm, right-sided (diameter 32 × 27 mm)
**Bypass type**	Replacement ECA-ICA	Replacement ECA-ICA	Replacement ECA-MCA
**Intraoperative Bypass flow**	47 cc/min	160 cc/min	49 cc/min
**MRS score**			
Preoperative	1	3	2
Postoperative 7 days	4	5	2
Postoperative 30 days	4	4	0
Postoperative 90 days	1	3	0
Final follow-up (months)	1 (35 months)	1 (30 months)	0 (36 months)
**complications**	Transient left hemiparesis	Transient left hemiparesis, epidural hematoma, evacuated	none
**Surgery time**	9 h 50 min	7 h 26 min	7 h 17 min
**SEcl anastomosis time**	13 min	14 min	12 min
**Patency**	no	yes	yes
**Flap retrieval**	0	0	+

MRS: modified Ranking Scale, ECA: external carotid artery, ICA: internal carotid artery, MCA: middle cerebral artery

## Patient 1

A 61-year-old female presented with headache, ptosis and progressive diplopia due to right-sided cranial nerve III and IV palsy (MRS1).

**Fig. 2:**
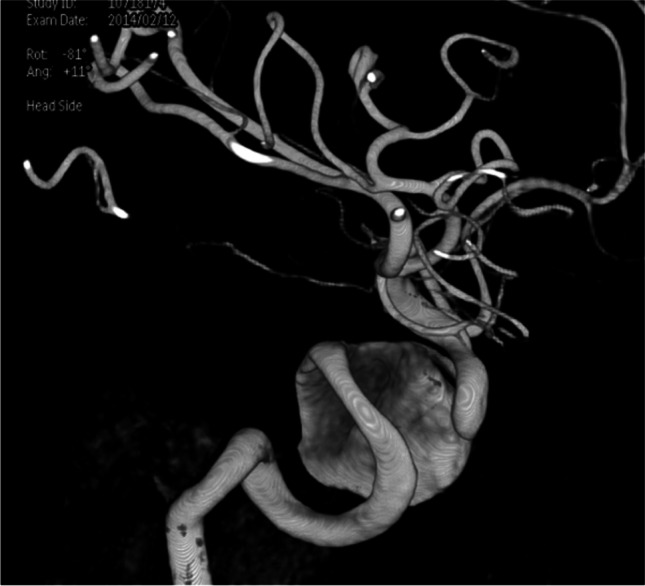
Patient 1: 3D DSA of right-sided partially thrombosed internal carotid artery aneurysm

A CTA scan showed a partially thrombosed right-sided giant ICA aneurysm in the cavernous sinus (carotid siphon aneurysm, dome diameter 2.8 cm) partially thrombosed, with local compression on the right cranial nerve III and IV (Fig. 2). Temporary balloon occlusion (TBO), performed in the referring hospital, was not tolerated due to a venous delay of 2 s. The multidisciplinary team decided that treatment was indicated, and as endovascular treatment was considered not feasible due to the partial thrombosis and proximal and distal stenosis of the ICA, an EC-IC bypass from the right external carotid artery (ECA) to the right distal intracranial carotid artery (ICA) with subsequent occlusion of the ICA was advised.

As the BOT was considered to be not tolerated, an EC-IC replacement bypass with subsequent occlusion of the right ICA was multidisciplinary decided as the best treatment option.

Surgical procedure: A right-sided pterional transsylvian craniotomy was performed.

After dural opening the right ICA, proximal middle cerebral artery (MCA) and anterior cerebral artery (A1) were exposed and small caliber posterior choroidal branch and posterior communicating artery (PCOM) were identified.

The cranial skin incision was then extended to the neck. The external carotid artery (ECA), ICA and common carotid artery (CCA) were located and exposed, and the ECA was prepared for proximal anastomosis construction. This anastomosis was constructed with running sutures during a temporary occlusion of the external carotid artery (ECA) for 24 min. After completion, there was high outflow (cut end out flow 200 cc/min) and a temporary clip was placed on the VSM, 5 cm from the anastomosis after insertion of heparin solution.

The SEcl was prepared for the distal, intracranial anastomosis on a separate operation table in ten minutes conform protocol using two stitches (Prolene 8.0).

The SEcl with mounted VSM was smoothly inserted at the ICA–MCA junction, and the SEcl anastomosis was lased. The duration of this procedure was 3 min.

The anastomosis was patent with immediate substantial reflux. However, upon inspection of the laser catheter no flap was observed. Given the good reflux and patency of the anastomosis, it was decided to continue with the construction of the vein-to-vein anastomosis of the bypass using running sutures (Prolene 8.0).

At completion of the bypass, no flow was detected using flowmetry (Transonic Systems,

USA). Since no flap was retrieved, a crosswise incision was made 1 cm above the distal SEcl anastomosis for inspection. For this, a temporary occlusion of the ICA was necessary (2 × 1 min). Some debris including the flap was removed. After this, a strong reflux was obtained from the ICA through the anastomosis as well as through the bypass.

The bypass incision was closed with 3 stitches (Prolene 8.0).

The right ICA was now permanently closed with two Mersilene 1.0. Flowmetry showed a flow of 47 cc/min through the bypass in the final stage of closure. Direct postoperative, the patient was hemiparetic on the left arm and leg.

**Fig. 3:**
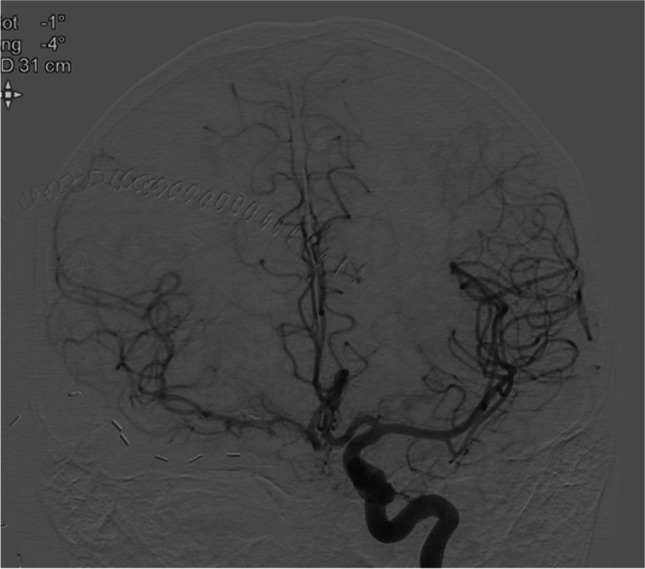
Patient 1: DSA showing complete left to right overflow after right ICA occlusion

CTA angiography showed delayed perfusion of the right hemisphere and occlusion of the aneurysm but also that the bypass was thrombosed as was confirmed by DSA (Fig. 3).

The occlusion was considered to be the result of competing flows between the bypass and he ACOM and leptomeningeal collaterals and not as a device-related adverse event (DSMB).

After 3 months, she was nearly recovered with only a neglect of her left arm and follow-up after 22 months showed that also the neglect has recovered with only the cranial nerve III and IV palsy as preoperative (MRS 1). She continued her work 6 months after surgery.

## Patient 2

The second patient was a 62-year-old female referred by another academic center where she was monitored for bilateral cavernous sinus ICA aneurysms. She previously was treated using a flow-diverting stent in the left ICA aneurysm, but the stent occluded leaving her with an occluded left ICA and complete left NIII palsy. Due to growth of the right ICA aneurysm, she presented with also a progressive disabling ptosis of the right eye which she kept open with a bandage.

**Fig. 4:**
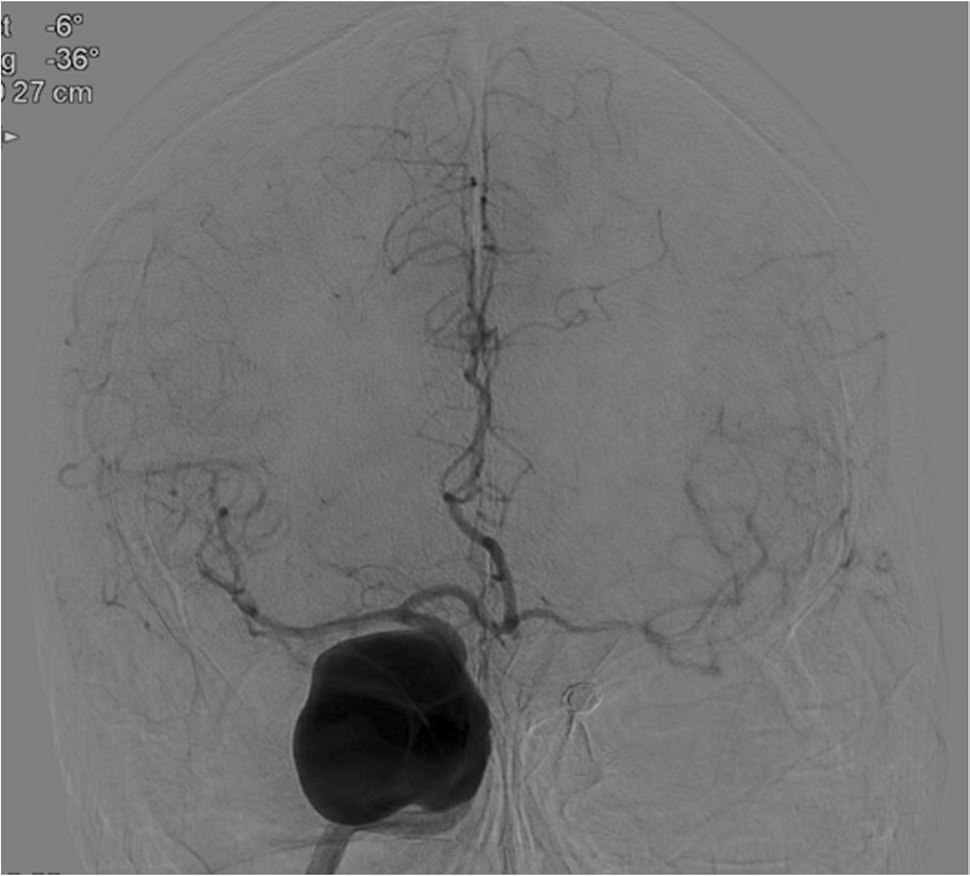
Patient 2: DSA of growing right internal carotid artery aneurysm

In addition, diplopia and visual field disturbances made her unable to walk without assistance (MRS 3) (Fig. 4).

DSA showed an occluded left ICA with a flow-diverting stent in situ and the left ICA aneurysm excluded from circulation. The left hemisphere was completely vascularized by leptomeningeal and circle of Willis collaterals from the right ICA and PCOM with minimal retrograde filling of the left ICA. Both MCA’s, the basilar artery and PCA’s and PCOMS were patent.

The local multidisciplinary team considered that treatment was mandatory and a that BOT was not informative. Endovascular treatment using stenting of the right ICA was not indicated considering her medical history of occlusion of the left-sided stent. A preferably non-occlusive high-flow bypass technique (ELANA) was advised taking the high blood flow needed after the intended occlusion of the right ICA into account.

Surgical procedure: First opening of the neck and exploring the common carotid artery for proximal control. After a frontotemporal craniotomy and microscopic dural opening, the Sylvian fissure was opened and followed medially in the depth until the ICA distal to the aneurysm was fully exposed (diameter 3.5 mm). The ICA–MCA complex was slightly twisted and pressed upward by the aneurysm.

The right VSM was harvested over a long trajectory (± 20 cm) and mounted on the SEcl with five interrupted micro-sutures (Prolene 8.0) in 6 min, and adequate intraluminal space was verified using the sizing catheter.

The donor ECA was prepared for proximal anastomosis construction. The anastomosis was constructed with running sutures (Prolene 8.0) during a temporary occlusion of the ECA for 19 min and showed high cut end outflow of 220 ml/min, and a temporary clip was placed on the VSM 5 cm distal to the anastomosis after insertion of heparin solution.

Consequently the SEcl and VSM were inserted and locked on the right ICA in 4 min under normotension with direct hemostasis. The laser catheter was inserted, and after two minutes vacuum, the laser is applied twice for 5 s, consecutively resulting in a strong backflow. Total anastomosis time was 14 min. At withdrawal, no “flap” was observed on the laser catheter; however, it was probably flushed away by the strong back flow.

The interposition vein end-to-end anastomosis was then constructed with running sutures (Prolene 8.0). Flowmetry showed a maximum flow through the bypass of 180-190 cc/min during test closure of the RICA and stabilized at 160 ml/min. Next, the right ICA was closed in the neck permanently with Mersilene 1.0. During closing, the transonic flow in the bypass remained stable at 160 cc/min.

**Fig. 5:**
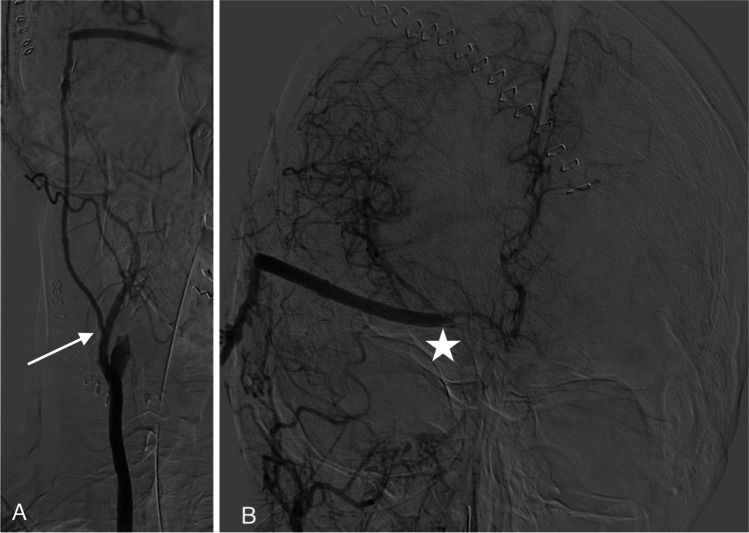
Patient 2:DSA of SEcl high flow EC-IC bypass after occlusion of the right internal carotid artery. Figure 5 b: Proximal anastomosis on the external carotid artery (arrow). Figure 5 b: Distal anastomosis (SEcl) on the distal tight internal carotid artery (asterisk)

Several hours postoperative, after initial good recovery she deteriorated with an hemiparalysis left due to an epidural hematoma that was evacuated immediately and the bone flap left out. CTA showed that the bypass was patent, and the aneurysm and ICA occluded. Postoperative DSA after 5 days demonstrated that the bypass was patent (Figs. 5a and 5b). During her stay of ten days, the paralysis recovered to MRC 4 + .

After 3 months rehabilitation, the hemiparesis was fully recovered and the ptosis for 90% (MRS 2). After 1 year, the bone flap was replaced. At follow-up after 30 months, the MRS scored 1.

**Fig. 6:**
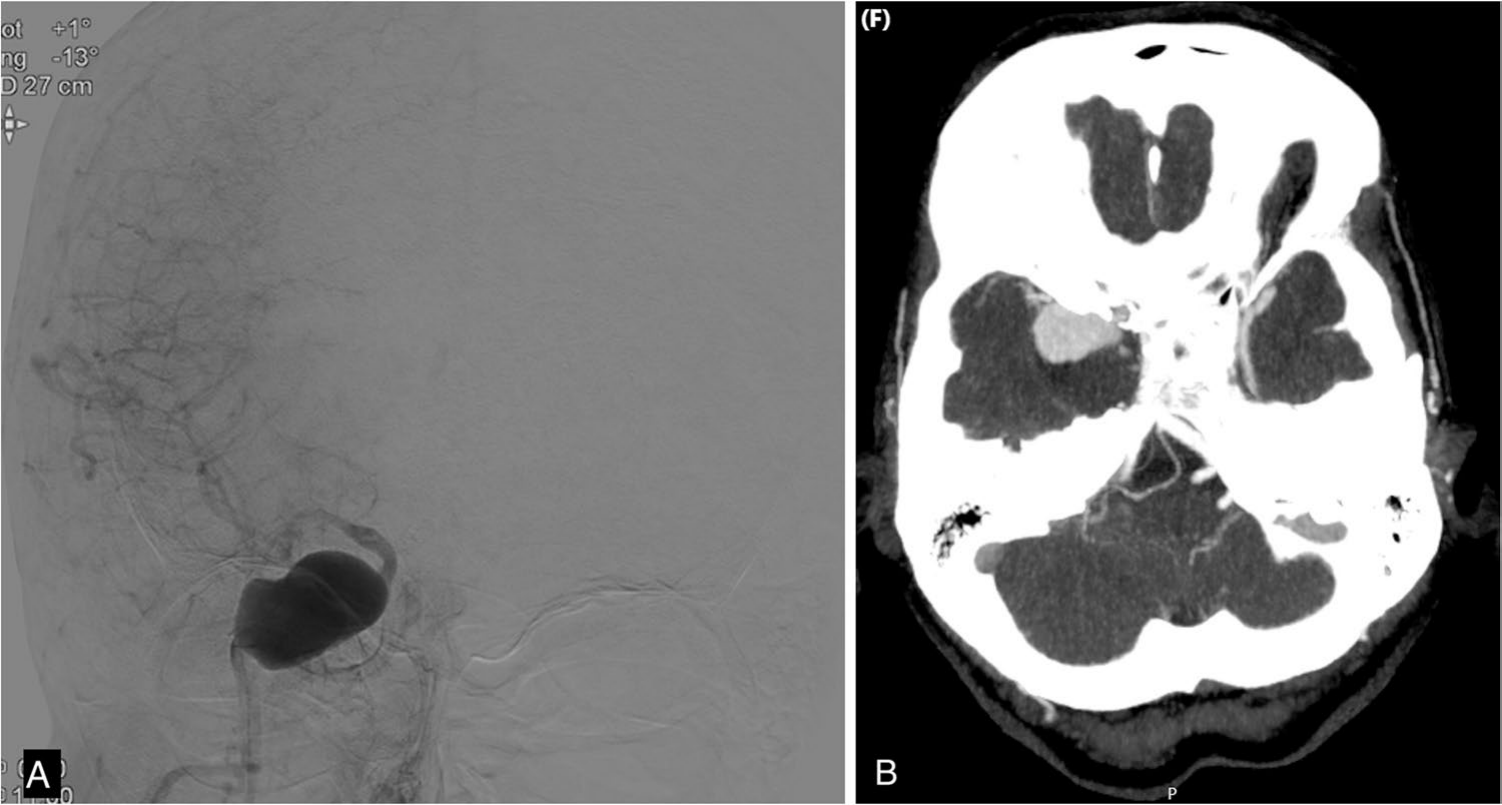
Patient 3: **a**: DSA of partially thrombosed of aneurysm of right internal carotid artery **b**: CTA

**Patient 3** (Fig. 6, Video 1, Supplemental Digital Content 1, synopsis of the SEcl bypass procedure).

A 66-year-old female patient was referred with diplopia and decreased visual acuity due to progressive ptosis that limited her in her activities (e.g., riding a bike) due to a very large right-sided internal carotid aneurysm (MRS 2).

Neurological examination showed a slight paresis of the abducens nerve on the right side with corresponding diplopia, as well as a 50% ptosis.

CT angiography and DSA showed a partly thrombosed giant aneurysm of the right ICA (32 × 27 mm) with stenosis at the inlet of the aneurysm. TBO previously performed showed a venous delay of 1 s.

The local multidisciplinary team considered that treatment was mandatory, and a high-flow replacement bypass was preferably indicated followed by proximal occlusion of the ICA.

Surgical procedure: right pterional approach of the ICA and proximal MCA. The ICA was identified with vision on the aneurysm bulging through the dura at the temporal media fossa. Since the ICA was arteriosclerotic, the distal SEcl anastomosis site was decided to be at the proximal MCA. Intraoperative flow of the proximal MCA was 75 cc/min with an external diameter of 3.1 mm. The skin incision was then extended to the CCA, and the ICA and ECA were identified to ensure also proximal control of the aneurysm. The right VSM was harvested. The proximal, hand-sutured anastomosis was constructed with the graft during temporary clipping of the ECA during 21 min with using running sutures (Prolene 8.0), and a temporary clip was placed on the proximal donor vein after insertion of heparin solution.

The distal VSM was mounted on the SEcl and secured with 4 interrupted micro-sutures (Prolene 8.0) for 7 min, and adequate intraluminal space was verified using the sizing catheter.

Subsequently, the SEcl and VSM were inserted and clipped on the MCA in 3 min under normotension with direct hemostasis. The laser catheter was then inserted, and after two minutes vacuum, the anastomosis was lasered, resulting in a strong backflow. Total anastomosis time was 14 min. At withdrawal of the catheter, a “flap” was retrieved. A temporal clip was placed on the distal bypass vein, and the interposition vein end-to-end anastomosis was constructed by running sutures (Prolene 8.0). Flowmetry showed a potential maximum flow of 60 cc/min during test closure of the right ICA. This meant a flow deficit of 15 cc compared to the measured MCA flow before bypass construction (75 cc/min). Taking the collateral capacity PCOM and ACOM into account, the bypass flow was considered to be sufficient for adequate flow replacement. The proximal ECA was then definitively occluded using Mersilene 1.0. During closing, the transonic flow remained stable at 49 cc/min.

**Fig. 7:**
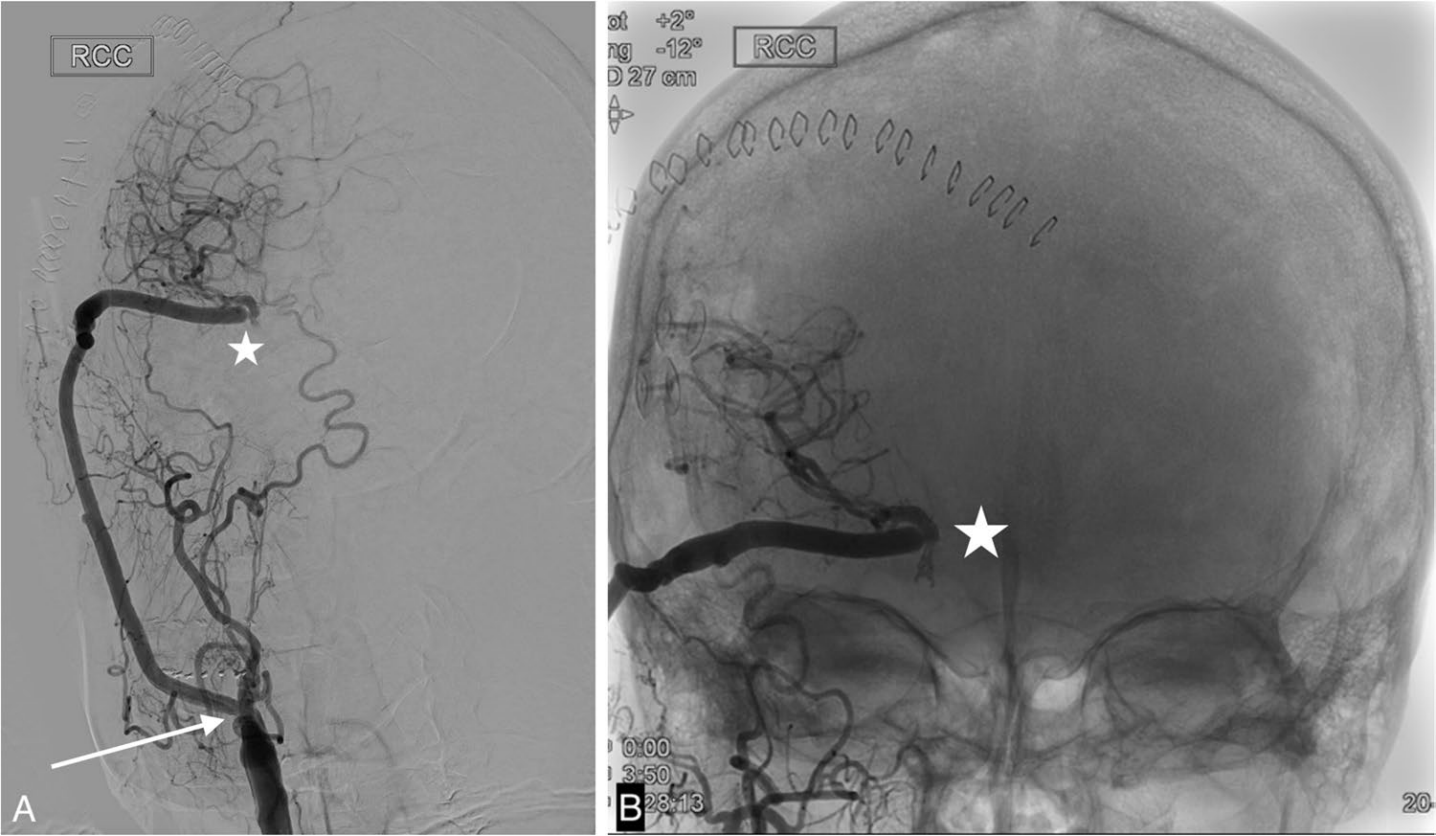
**a**: DSA of SEcl high flow EC-IC bypass after occlusion of the right internal carotid artery. Proximal anastomosis on the external carotid artery (arrow). Distal anastomosis (SEcl) on the middle cerebral artery (asterisk) **b**: Detail of distal anastomosis with clip in situ (asterisk)

Postoperatively the patient was neurologically intact. CT-angiography and DSA showed the occluded ICA and a patent bypass with no perfusion deficits (Fig. 7).

She was discharged in good condition nine days postoperatively (MRS 1).

At 30-day follow-up, she showed full recovery and only minimal residual facial asymmetry.

At 90-day follow-up, she did not experience any complaints, no neurological deficits could be observed (MRS 0) and this continued up to follow-up after 36 months. The aneurysm was completely removed from the circulation and the bypass remained patent by follow-up with CTA.

## Discussion

Although most large or giant intracranial aneurysms nowadays can be treated endovascularly, the neurosurgical treatment of those aneurysms that are not safely treatable by coiling or stenting is highly complex. In many cases, bypass flow replacement surgery still is preferable. Especially in high-flow bypass surgery, the ELANA technique is one of the options considering its non-occlusive characteristic. The present feasibility study describes the first-in-human application of the SEcl device in three cases.

Feasibility: In all cases, the application of the device, mounting the VSM and creating the anastomosis in 13, 14 and 12 min, respectively, proved to be much shorter than the creation of the historical ELANA anastomosis (90 min on the ICA) [20].^.^ It is noteworthy that the mean conventional anastomosis construction time in the COSS/ EC-IC bypass study (45 min) is longer than the construction times in the present study [18]. This underlines the feasibility of the SEcl technique in well-selected patient cohorts.

The main reason is that the placement of at least 8 stitches in the depth within the confined craniotomy window at the level of often the ICA is now no longer necessary. The clip replaces this tedious and time-consuming work. The hemostasis proved to be excellent, and no pseudoaneurysm formation was detected during follow-up.

The current SEcl technique is easier to perform than a conventional anastomosis at this depth, and the large differences in construction time underline that this novel SEcl technique also simplifies the technique in practice.

In two patients, the flap was not retrieved on the catheter tip or found at the anastomosis site. In our previous experience, this is not of clinical significance as in most of the cases the flap is flushed out. Nevertheless, the flap retrieval remains an important issue in laser-assisted anastomosis creation.

Safety: Severe adverse events occurred in two out of three patients (patients 1 and 2): In patient 1, an infarct of the watershed type occurred and it took 3 months to recover. The flap was manually removed after temporally opening of the bypass. The occlusion of the bypass in this patient could be the result of this manipulation and as a consequence damaging the endothelium. In our opinion, however, the competing flow from own collaterals caused the flow conducted through a venous conduit (VSM) to be low and consequently thrombosis of the bypass at the anastomosis site. In patient two, the flap flushed away from the catheter, however no ischemia occurred. These findings also underline the most difficult and delicate phase in the (S) ELANA technique: retrieval of the flap. In our previous studies, catheter flap retrieval occurs in 86% of the cases. In patient 2, a postoperative epidural hematoma occurred and was evacuated immediately and the boneflap removed. The bypass remained patent. The patient recovered to her preoperative state (MRS 3) within 3 months. These serious adverse events were procedure-related but considered as not related to the clip device itself.

Outcomes were favorable in all patients: All aneurysms were removed from the circulation, and final long-term MRS scores (35 months, 30 months, 36 months) were stable in patient 1 and improved in two patients (patient 2 and 3).

The occlusion of the bypass in patient 1 underlines the complexity of the treatment of this patient cohort: Although preoperative TBO showed a venous delay of 2 s, the bypass flow was competitive with leptomeningeal and circle of Willis collaterals in this patient. The flow through this venous bypass was only 47 cc/min and the bypass occluded. Postoperatively, the MRS score increased to 4 but was recovered to the preoperative state within 3 months. This phenomenon again subscribes the ambiguity of the use of venous delay of 2 s in the TBO [7]. In retrospect, both patient 1 as patient 3 could have been treated with a STA-MCA bypass surgery considering the measured flows in the venous bypasses (47 cc/min and 49 cc/min). At that time, however, we decided to be on the safe side by choosing for a large caliber non-occlusive bypass. However, this choice may also have had the counteracting result of thrombosis of the bypass (patient 1). Therefore, in future decisions it is mandatory to improve patient selection for this type of bypass surgery. For evaluating the flow replacement demand, techniques such as flat detector computed tomography cerebral blood volume (FD-CBV) imaging, single-photon emission computed tomography (SPECT), MRI BOLD or MRA could serve as adjunctive techniques in patients with large or giant intracranial aneurysms for the evaluation of therapeutic occlusion of the ICA with or without large-, medium- or low-size bypass surgery [8]. Consequent intraoperative evaluation of the capacity of the STA as donor using cut flow measurements could be helpful in the decision of which type of bypass should be used. Further studies on cerebral hemodynamics as preoperative and intraoperative tools are important to improve the assessment of the collateral capacity in these patients and consequently improve therapy selection.

Patient Outcome: At last follow-up, all patients reported to be very satisfied with the operation. They were able to return to their previous life and participation in society.

Patients diagnosed with an unruptured aneurysm are known to have a significantly reduced quality of life. Intuitively, part of the reported positivity and satisfaction of our patients can be explained by successful treatment and occlusion of the aneurysm. Interestingly, however, several studies showed that the quality of life in these patients does not improve much after treatment with occlusion of the aneurysm and even shows a first-year decrease when treated with clipping or bypass operation [1, 2]. Our patient outcomes, also within the first year of follow-up, were therefore encouraging.

Although the SEcl bypass technique shortens total surgery time, the complexity of these difficult-to-treat aneurysms still makes open surgery a challenging and time-consuming procedure. Avast reduction in bypass construction time can theoretically also reduce the OR time. In this study, the total surgery times were not reduced despite the shorter bypass construction time (mean 490 ± 86 min).

In addition, improvements of interventional technology techniques and consequent decreasing numbers of flow replacement bypasses being performed also diminish the need for ELANA centers. However, it remains of great importance that vascular neurosurgeons continue to master bypass techniques and maintain creativity and open-mindedness in studying and adopting new technologies as interventional neuroradiologists do.

An evident limitation of this pilot study is the small number of patients. Since the technique is a last-resort flow replacement treatment option for patients with complex cerebral aneurysms, and nowadays vein bypass grafts are rarely warranted, the number of patients is very limited. This remains a challenge for further investigation of the technique. Especially in flow augmenting bypass surgery in ICA occlusion, the non-occlusive character of the SEcl device could diminish the risk of perioperative ischemia [9, 10, 12, 13, 19]. In the well-known COSS trial, the EC-IC bypass surgery arm was associated with a 12–15% risk of perioperative ipsilateral infarction [14]. The mean temporal occlusion time was 45 min but extended up to 90 min. Beside more advanced preoperative diagnostics such as flat detector computed tomography cerebral blood volume (FD-CBV) imaging, single-photon emission computed tomography (SPECT), MRI BOLD or MRA, a non-occlusive anastomosing technique using the SEcl technique could be advantageous for those patients. Adapting the SEcl device to be used on smaller arteries (1.5- 2.6 mm) is now under investigation in our institute and preliminary results are very promising.

Finally, as a consequence of the years of development of the SEcl technique, the application of this concept is currently under investigation for application in cardiac bypass surgery [3, 15, 16]. Potential advantages of the sutureless ELANA technique may result from its non-occlusive nature and herewith preventing downstream ischemia of heart tissue. In addition, the SEcl induces direct hemostasis. Finally, the ELANA heart bypass could enable a minimally invasive approach of the cardiac bypass surgery, which is performed on a beating heart and thus could eliminate the need for a heart–lung machine as well as associated risks and complications. As yearly 900.000 patients undergo a coronary artery bypass grafting worldwide, further development of the present SEcl technique for cardiosurgical application could have major clinical impact.

## Conclusion

The novel SEcl technique is feasible with acceptable safety parameters showing a favorable outcome with successful treatment of the aneurysm and better or equal postoperative MRS score in three very difficult-to-treat patients that could not be treated otherwise. However, conscientious patient selection remains crucial in this type of neurosurgery.

## Supplementary Information

Below is the link to the electronic supplementary material.

Supplementary file1 (MP4 16793 KB)

## Abbreviations

ELANA: Excimer laser-assisted non-occlusive anastomosis
Secl: ELANA Clip device
ICA: intracranial internal carotid artery
SEsl: ELANA slide device
DSA: digital substraction angiograpy
CTA: computed tomographic angiography
MRA: magnetic resonance angiography
BOT: balloon occlusion test
VSM: large saphenous vein
DSMB: Data Safety Monitoring Board
ICG: indocyanine-green angiography
MRS: modified Rankin score
MCA: proximal middle cerebral artery
A1: proximal anterior cerebral artery
PCOM: posterior communicating artery
ECA: external carotid artery
FD-CBV: computed tomography cerebral blood volume
